# A New Click Beetle Genus from Southern Chile: *Llanquihue* (Coleoptera, Elateridae, Elaterinae, Pomachiliini)

**DOI:** 10.1673/031.008.3701

**Published:** 2008-05-09

**Authors:** Elizabeth T. Arias

**Affiliations:** Essig Museum of Entomology, University of California, Berkeley, 210 Wellman Hall, Berkeley, CA 94720

**Keywords:** *Deromecus*, Pomachiliini

## Abstract

*Llanquihue*, a new genus of Elateridae from Southern Chile, is here described and illustrated with 2 species: *Llanquihue vittipennis* (Candèze) new comb., and *L. carlota* sp. nov. The genus *Llanquihue* belongs to the subfamily Elaterinae and to the tribe Pomachiliini.

## Résumé

Se describe e ilustra Llanquihue, género nuevo de Elateridae con dos especies: *Llanquihue vittipennis* (Candèze) new comb., and *L. carlota* sp. nov. El género *Llanquihue* pertenece a la subfamilia Elaterinae y a la tribu Pomachiliini.Se describe e ilustra *Llanquihue*, género nuevo de Elateridae con dos especies: *Llanquihue vittipennis* (Candèze) new comb., and *L. carlota* sp. nov. El género *Llanquihue* pertenece a la subfamilia Elaterinae y a la tribu Pomachiliini.

## Introduction

The fauna of Pomachiliini in Chile is formed by the following genera: *Deromecus* (Solier 1851), *Gabryella* (Arias 2001a), *Lynnyella* (Arias 2001b), *Alyma*Arias (2004), *Mecothorax* (Solier 1851), *Pomachilius* (Eschscholtz 1829), *Podonema* (Solier 1851), *Medonia* (Fleutiaux 1907), *Pseudoderomecus* (Fleutiaux 1907), and *Sofia* (Arias 2005). In 1851 Edgard Solier, a French naturalist was the first to study Chilean Elateridae. Solier (1851) created the genus *Deromecus* with 8 species. Later, in 1900, Candèze, a Belgian naturalist, described *Deromecus vittipennis.* However, this species does not share the generic characters of the genus *Deromecus.* A new genus *Llanquihue* is proposed to include the species *vittipennis* (Candèze) new comb., and *carlota* sp. nov.

**Figure 1.**
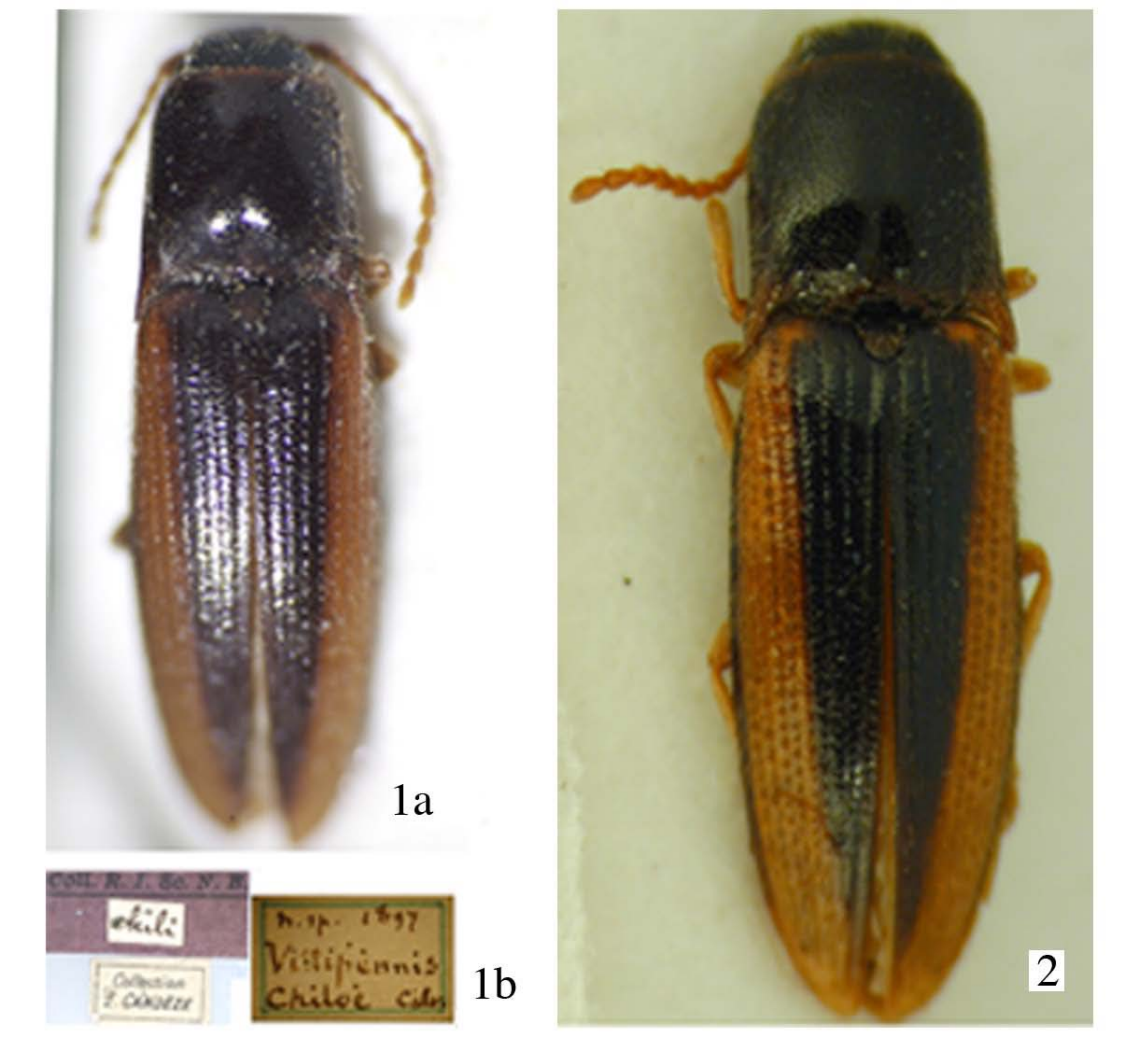
(a) Type of Deromecus *vittipennis* (Candèze). (b). Type labels of *D. vittipennis.* Figure 2. Type of *Llanquihue carlota* Arias

## Materials and Methods

Measurements were made with a calibrated ocular micrometer. The total body length was measured (mm) from the frontal margin to the apex of the elytra, and elytral width was the maximum width of the elytra, when both sides were in focus. Indices are indicated as follows. 1) The eye index an index of eye prominence was obtained by subtracting the interocular head (frons) width from the maximum width of the head across the eyes and dividing the result by the maximum head width. 2) Pronotal elytral index was obtained by dividing the length of the pronotum by the length of the elytra, the pronotal elytral index is used here because it gives a general idea of how big the pronotum is compared with the elytra. 3) The pronotal index was obtained by dividing the length of the pronotum by its width (Calder 1996). 4)

**Figure 3.**
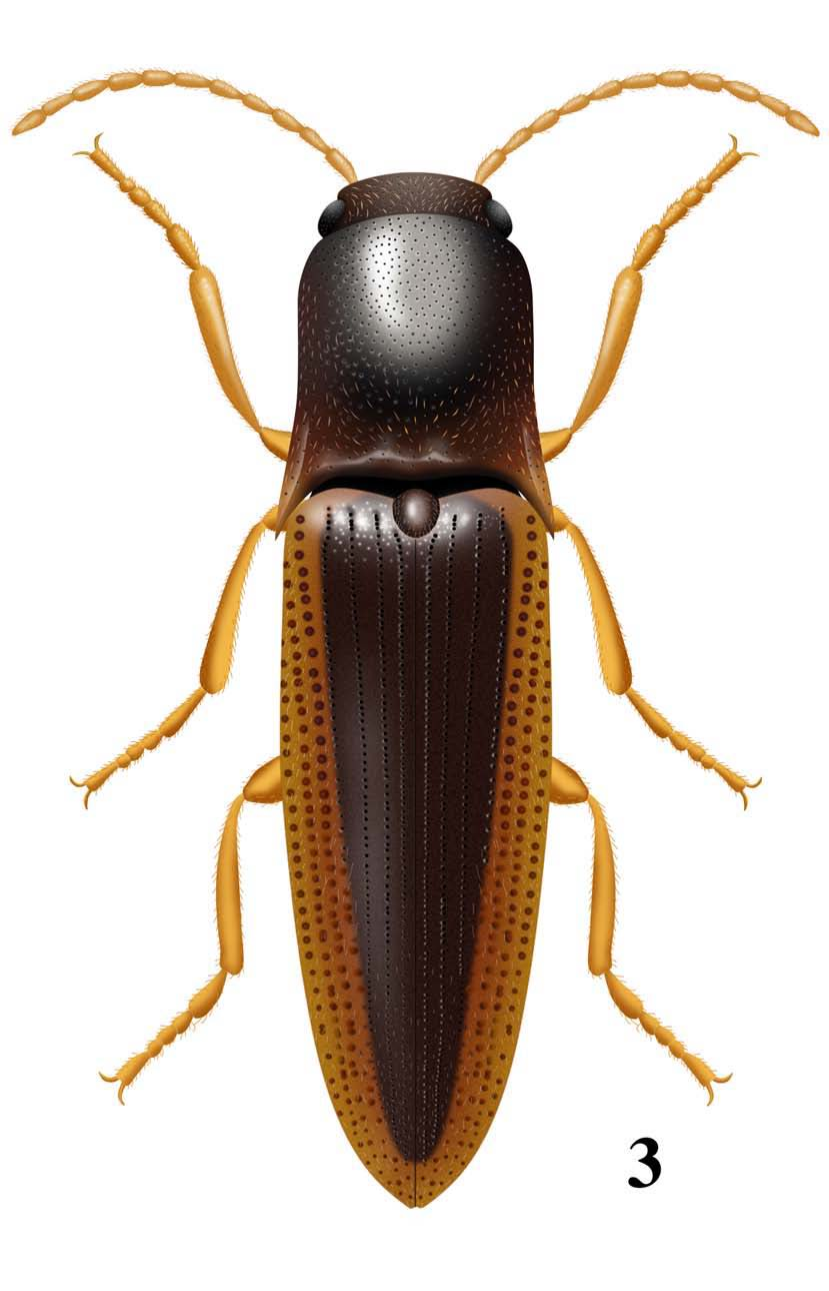
*Llanquihue carlota*, dorsal habitus. Illustration by Nancy V. Arias.

**Figure 4.**
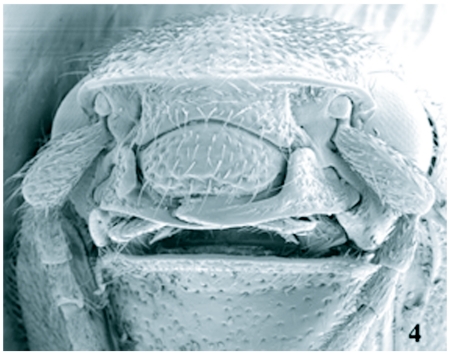
Scanning electron micrograph of frontal head of *Llanquihue carlota.*

Antennomere proportion was measured as the length of antennomeres 2 through 11 as 1/100th of the total antennal length. This is not measured for antennomere 1 because it is curved and hard to measure. 5) Total body length was measured from dorsal view including the head. 6) Tarsomere proportion gives the lengths of the tarsomeres as 1/100th of the total tarsal length.

Specimens from which the genitalia were to be removed were first relaxed overnight in warm water with a few drops of soap added. For examination of male genitalia, the last abdominal segment was removed and placed in water with a few drops of soap in a Petri dish and left over night. Then, genitalia were removed and glued to a point card on its lateral side with balsam, and placed on the pin under the specimen. Becker (1958) was followed for female genitalia examination.

Drawings were made using a camera lucida on a dissecting scope Leica MZ7. All dates in the records given were converted to a standard format of day.month.year, with the month given in Roman numerals. Places and names in the recorded labels are the original spellings.

Museums and institutions that contributed to this work are indicated in the acknowledgements and in the text by the acronyms in brackets (Arnett et al 1997), excluding [ETA] author's collection. Type specimen repositories are also indicated in descriptions. Type material will be deposit at the Museo Nacional de Historia Natural, Santiago Chile [MNNC].

**Figure 5.**
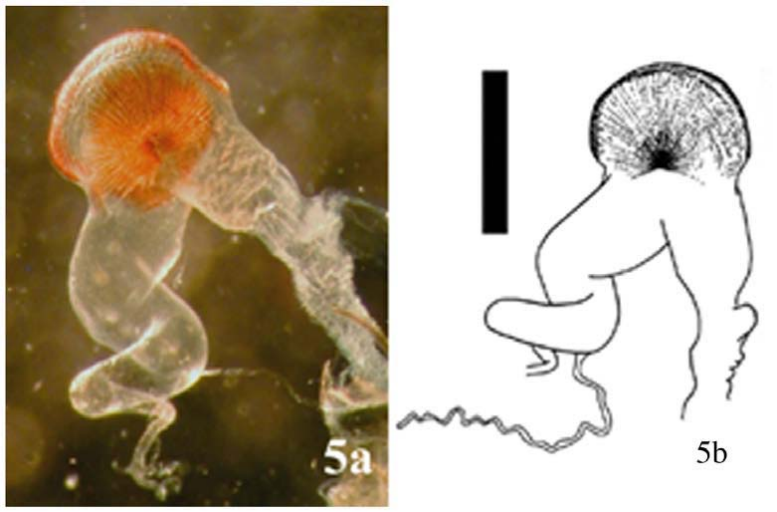
(a). Female genitalia of *Llanquihue vittipennis* n. comb, photograph; (b), illustration (Nancy V. Arias). Scale bar = 1 mm.

**Figure 6.**
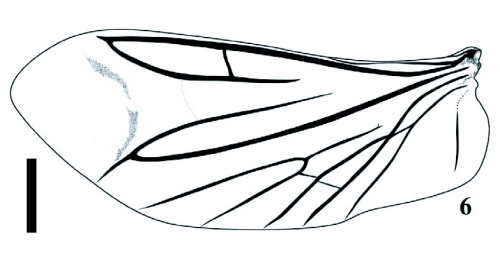
Wing venation illustration of *Llanquihue carlota* (Nancy V. Arias). Scale bar = 1 mm

**Taxonomy Llanquihue Gen. Nov. (Figures 1a, 2, 3) Type species *Deromecus vittipennis* Candèze 1900:91.**
**Description**
  Body stout (Figures 1a, 2, and 3); general body color brown, dark brown, or reddish brown, blackish, a long darker stripe on the elytral suture, extended side ways covering almost all elytral surface, broader anteriorly and narrower towards elytral apex.
**Head**
  Declivous, punctate; vestiture long, pale or gold, semi-erect, or semi-decumbent, sparse or dense; frontoclypeal region sloping to base of clypeus, not intercepting clypeus; clypeus, narrow at center; frontal carina complete across front of frons, slightly protruded (Figure 4); eyes small, [eye index: 0.25]; labrum exposed, tall, vertical, curved anteriorly; antennae with eleven antennomeres, antennomere ten reaching apex of posterior angles; antennomere fourth through antennomere eleven serrate; vestiture semi-erect; mandibles bidentate, maxillary and labial palps with apical segments securiform.
**Prothorax**
  Not convex medially or anteriorly; [pronotal index: 0.94–1.0]; narrowed anteriorly to receive head; lateral margins entirely carinate, almost all its length straight, except at posterior angle base; inclined mesodorsally; lateral carina directed ventrally, visible only posteriorly; pronotal lateral margin joining pronotosternal suture apex; pronotal punctures puncticulate, or punctate; pronotal basal area declivous to prescutum; pronotal basal margin curved; small notch at each side of base of pronotum, near base of posterior angles; prescutum notch small, V-shaped; posterior angles small, acute, unicarinate, straight or slightly divergent; prosternum longer than wide, convex; pronotosternal lobe bent, border thick; antennal groove present, carinate in pronotosternal hypomeral side; pronotosternal suture thickened giving a double appearance, marginate at procoxal margin, curved at procoxal margin; prothoracic sternite around procoxae marginate; prosternum with furrow along pronotosternal suture, ⅔ of pronotosternal suture length; pronotosternal spine more or less horizontal with a ledge immediately after procoxae; procoxae globular, marginate.
**Scutellum**
  Tongue shape; mesosternal cavity more or less oval, deep; posterior margin of mesosternal cavity extending shortly in distance posteriorly; mesocoxae longer than wide; mesocoxal cavity deep, open to mesepimeron and mesepisternum; mesosternum and metasternum separated by distinct external suture.
**Elytra**
  Parallel-sided on half of its anterior length; 2.5–3.1X pronotal length; striate, striae with pits tear shape and or circular; apex truncate or dentate.
**Metathoracic wings**
  Apical portion of the wing with 2 soft plates; r4 present, wedge cell present width 3.78X its length, joint of MP_3_+ MP4+CuA1 slightly bifurcate at origin; radial cell 4X its width, MP_4_ + CuA_1_ connected with CuA_2_, base of wedge cell not contiguous with connective vein between MP_4_ + CuA_1_ and CuA_2_ (Figure 6). Metathoracic coxal plate widest region closest to medial body line; with setae semi-decumbent, gold.
**Legs**
  Yellowish; femur globate yellow; tarsomeres 1 through 4 decreasing in length distally, tarsomere 3 cordate, tarsomere 4 very small in size compared with other tarsomeres.
**Abdomen**
  Ventrites with punctures, last ventrite angulate.
**Genitalia**
  Female: vagina without sclerotized internal structures; strongly elongate and enlarged towards the apex, slightly wrinkled at apex; before bursa, 2 globular gland similar in shape; bursa copulatrix 0.73 mm in diameter; globular, with two sclerotized fan shaped structures with teeth alternating between long and short and another long sclerotized structure dorsal; after bursa spermathecal gland with a minimum of 2 small sclerotized structures comb-shaped, and one delicate nonsclerotized sinuate structure attached (Figures 5 a, b). Male: parameres not reaching apex of aedeagus, wider than width of aedeagus (Figures 13–14).
**Distribution**
  Llanquihue province, and Chiloé island, Region X of Chile. Temperate Rainforests.
**Biology**
  Adult specimens were collected during spring season. Species from this new genus do not have distinctive sexual dimorphism and specimens need to be dissected to discriminate between males and females.
**Etymology**
  The designation of this genus is after the *Llanquihue* Lake that in Mapudungun means immerse placed. The Llanquihue Lake is the second largest lake in Chile, with an area of 860 km^2^.
**Remarks**
  The new genus *Llanquihue* belongs to the subfamily Elaterinae (Leach 1815) because the adults are characterized by (Calder 1996): head distinctly convex anteriorly; frontal carina usually complete across front between eyes or incomplete medially; antennae nearly always serrate; prosternal spine usually longer than the procoxal diameter; mesocoxal cavity open to both mesepimeron and mesepisternum; hind wing with a short radial cell; vein MP4 with apparent cross vein to CuA2. The new genus *Llanquihue* belongs to the tribe Pomachiliini (Candèze, 1878) because adults are characterized by: presence of a frontal carina across the frons (Platia 1994); prosternum with prosternal suture thickened on the hypomeral side giving an appearance of a suture double (Hayek, 1990).

**Figures 7–8.**
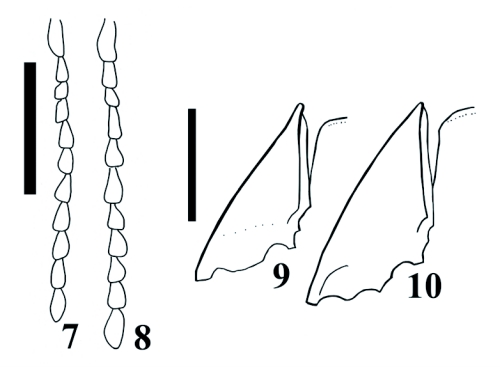
Antennomeres of *Llanquihue* species. 7, *L.* *vittipennis;* 8, *L.* *carlota*. Scale bar = 0.5 8 mm. Figures 9–10. Pronotosternal hypomera of *Llanquihue* species. 9, *L.* *vittipennis;* 10, *L.* *carlota*. Scale bar = 1 mm.

**Figures 11–12.**
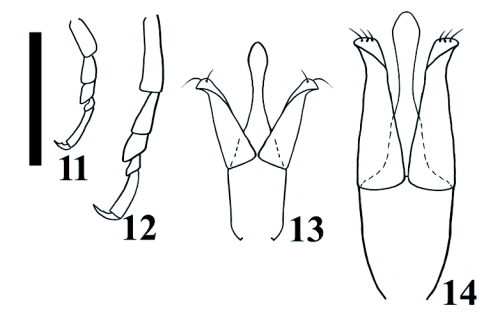
Tarsomeres of *Llanquihue* species. 11, *L.* *vittipennis;* 12, *L.* *carlota*. Scale bar = 0.5 mm. Figures 13–14. Male genitalia of *Llanquihue* species. 13, *L.* *vittipennis;* 14, *L.* *carlota*. Scale bar = 0.5 mm.

**Figure 15.**
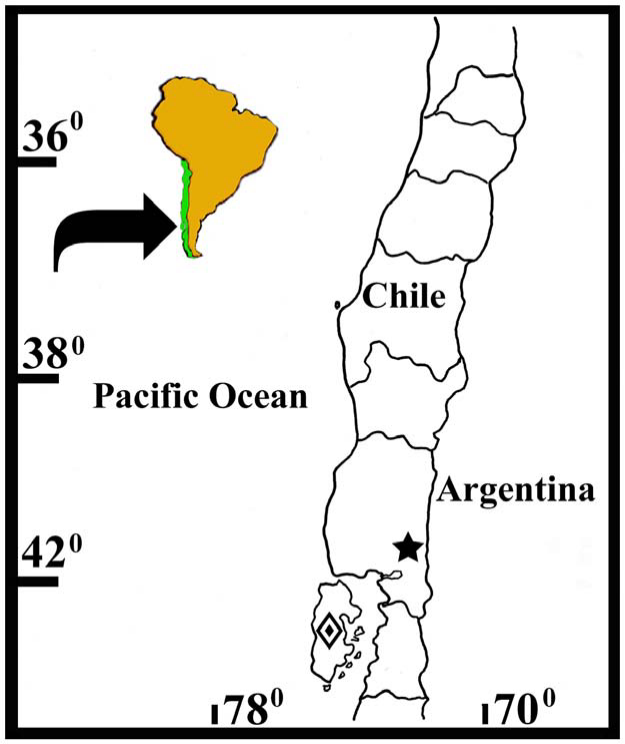
Geographic distribution of *Llanquihue* species. 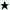, *L.* *vittipennis;* 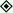, *L.* *carlota.*

***Llanquihue vittipennis* (Candèze), new comb. (Figures 1a, 5, 7, 9, 11, 13, 15) *Deromecus vittipennis* Candèze, 1900: 91.**
**Description**
  Body stout (Figure 1a); total body length 6.3 mm including head (head 0.14 mm), width 2.84 mm measured at widest point; general body color dark brown, blackish; integument semi-shiny; vestiture semi-erect, gold; [pronotal elytral index: 3.19].
**Head**
  Black; labrum 1.66X as long as wide; antennomeres 3–11 serrate, antennomere 2 bigger in size than antennomere 3 [antennomere proportion: 8.3-7.0-8.3-11.0-9.7-10.5-11.0-10.5-10.5-13.2] (Figure 7).
**Prothorax**
  Brown reddish; integument semi-shiny; punctate, punctures separated by one or 2 own diameters; [pronotal index: 0.94]; posterior angles straight; distinctive longitudinal impression at pronotal base; prosternum convex; pronotosternal hypomeron rugulose; antennal groove carinate on pronotosternal hypomeral side, (Figure 9); procoxae separated by 0.94X procoxal diameter; pronotosternal spine 1.1X procoxal diameter.
**Scutellum**
  Dark brown; 1.2X as long as wide; mesocoxae separated by 0.45X mesocoxal diameter; posterior margin of mesosternal cavity extending posteriorly 0.18X mesocoxal diameter.
**Elytra**
  Pits dense; anterior pits not surrounded by a darker area; elytral anterior border carinate; vestiture dense; apex truncate.
**Leg**
  Yellowish; vestiture light yellowish; [tarsomere proportions: 38.6-15.8-15.8-3.5-26.3] (Figure 11).
**Male genitalia**
  Aedeagus 0.53 mm long, 0.27 mm wide, as Figure 13.
**Material Studied**
  Holotype. Male: 0.53 mm in length, 0.27 mm in width. *Deromecus vittipennis* (card name hand written, border of card with a green line). Collection E. Candèze, Coll. R. I. Sc. N. B. (Figure 1b) [ISNB]. When Candèze designated types, he drew a color line on the label, depending on the region the animal was from (Hayek 1974). Other material studied. One male 528 Chiloé [MNNC]; 2 females Chiloé [MNNC]; male, Chiloé, Candèze [MNNC].
**Biology**
  There is no other currently available information on the biology of this species.
**Distribution**
  Chiloé island. X Region (Figure 15).
**Remarks**
  *Llanquihue vittipennis* can be recognized by its semi-shiny brown reddish punctate pronotal integument, gold vestiture, and posterior angles straight.

***Llanquihue carlota* sp. nov. (Figures 2, 3, 4, 6, 8, 10, 12, 14, 15)**
**Description**
  Body stout (Figure 2 and 3); length 6.07 mm including head (head 0.4 mm), width 1.84 mm measured at widest point; color brown; integument shiny; vestiture semi-erect, gold; [pronotal elytral index: 3.0].
**Head**
  Brown; labrum 1.56X as long as wide; antennomeres 3 through 11 sub-serrate, antennomere 2 bigger in size than antennomere 3, [antennomere proportions: 9.9-6.6-10.8-9.1-9.9-9.9-9.9-11.5-9.1-13.3], (Figure 8).
**Prothorax**
  Dark brown; integument shiny; [pronotal index: 1.0]; puncticulate; posterior angles slightly divergent; distinctive longitudinal impression from pronotal base to middle; prosternum convex; pronotosternal hypomeron punctate; antennal groove carinate on pronotosternal hypomeral, (Figure 10); procoxae separated by 1.0X procoxal diameter; pronotosternal spine 1.0X procoxal diameter.
**Scutellum**
  Dark brown; 0.88X as long as wide; mesocoxae separated by 0.75X mesocoxal diameter; posterior margin of mesosternal cavity extending posteriorly 0.45X mesocoxal diameter.
**Elytra**
  Pits dense, anterior pits surrounded by a darker area; elytral anterior border carinate; vestiture sparse; apex truncate.
**Leg**
  Vestiture light yellowish; [tarsomere proportions: 36.5-22.2-12.7-3.2-25.4] (Figure 12).
**Male genitalia**
  Aedeagus 0.79 mm long, 0.27 mm wide, as Figure 14.
**Material Studied**
  Holotype. Male: 6.07 mm in length, 1.84 mm in width. CHILE, Fresia, Llanquihue, XI. 1982. L. E. Peña [MNNC]. Paratypes here designated. 6 males, 2 females [MNNC]. Other material studied: 18 males, CHILE, Fresia, Llanquihue, XI. 1982. L. E. Peña [ETA].
**Biology**
  There is slight chromatic variation among specimens of this species, mainly posterior angles reddish or brownish reddish, and humeral area light yellowish or yellowish. There is no other currently available information on the biology of this species.
**Distribution**
  Llanquihue, X Region (Figure 15).
**Etymology**
  This genus is dedicated to my mother Carlota, who always took me, when I was young, to Southern Chile, especially to Llanquihue Lake, to know and appreciate nature. I have kept the feminine name on the species name.
**Remarks**
  *Llanquihue carlota* can be recognized by its dark brown semi-shiny puncticulate pronotal integument, pale vestiture, and posterior angles slightly divergent.

## Discussion

The genus *Llanquihue* differs from the genus *Deromecus* because of the following main characters: body stout; frontoclypeal region sloping to base of clypeus; frontoclypeal carina not protruding; clypeus narrow at center; base of pronotum with 2 notch; elytra with a dark stripe; wing venation lacks patch over rp1, whereas the genus *Deromecus* presents: body cylindrical; protruding frontoclypeal carina; clypeus broadly vertical, not narrow at middle, not intercepted by frontoclypeal carina; base of pronotum without a notch; elytra without a dark stripe; wing venation with a patch over rp1.

The phylogeny of the members of the subfamily Elaterinae is still uncertain. Future surveys and collections are needed to have a better understanding of the members of Elaterinae, such as the tribe Pomachiliini to which the genus *Deromecus*, and the new genus *Llanquihue*, belong.

## Editor's Note

Paper copies of this article will be deposited in the following libraries. Senckenberg Library, Frankfurt Germany; National Museum of Natural History, Paris, France; Field Museum of Natural History, Chicago, Illinois USA; the University of Wisconsin, Madison, USA; the University of Arizona, Tucson, Arizona USA; Smithsonian Institution Libraries, Washington D.C. U.S.A.; The Linnean Society, London, England.

## References

[bibr01] Arias ET (2001a). *Gabryella*, a new genus of click beetles from temperate South American Forests (Coleoptera: Elateridae).. *Contributions on Entomology International*.

[bibr02] Arias ET (2001b). *Lynnyella*, a new genus of click beetles from Central and South Chile (Coleoptera: Elateridae).. *Gayana*.

[bibr03] Arias ET (2004). A new genus of click beetle from temperate forests *Alyma* (Coleoptera: Elateridae: Pomachiliini).. *The Coleopterists Bulletin*.

[bibr04] Arias ET (2005). A replacement name for a Click Beetle Genus from Chile *Sofia* (Coleoptera: Elateridae).. *The Coleopterists Bulletin*.

[bibr05] Arnett RH, Samuelson GA, Nishida GM (1997). The insects and spider collections of the world.. *Flora and fauna Handbook N°11.*.

[bibr06] Becker CE (1958). The phyletic significance of the female internal organs of reproduction of Elateridae.. *Proceedings Tenth International Congress of Entomology*.

[bibr07] Candèze E (1878). Élatérides nouveaux.. *Annales de la Société Entomologique de Belgique*.

[bibr08] Candèze E (1900). Élatérides nouveaux.. *Annales de la Société Entomologique de Belgique*.

[bibr09] Calder AA (1996). Click beetles. Genera of the Australian Elateridae (Coleoptera).. *Monographs on Invertebrate Taxonomy*.

[bibr10] Eschscholtz JF (1829). Elaterites, Eintheilung derselben in Gattungen.. *Entomologische Archives*.

[bibr11] Fleutiaux E (1907). *Révision des Elateridœ du Chili.*. *Revista de Historia Natural*.

[bibr12] Hayek CMF von (1990). A reclassification of the Melanotus group of genera.. *Bulletin of the British Museum of Natural History (Entomology)*.

[bibr13] Platia G (1994). *Coleoptera Elateridae Fauna d'ltalia XXXIII.*.

[bibr14] Solier AJJ, Gay C (1851). Coleópteros elateroídeos, Zoología.. *Historia física ypolítica de Chile*.

